# Reduced Graphene Oxide Thin Film on Conductive Substrates by Bipolar Electrochemistry

**DOI:** 10.1038/srep21282

**Published:** 2016-02-17

**Authors:** Allagui Anis, Ali Abdelkareem Mohammad, Alawadhi Hussain, S. Elwakil Ahmed

**Affiliations:** 1 Dept. of Sustainable and Renewable Energy Engineering, University of Sharjah, PO Box 27272, Sharjah, United Arab Emirates; 2 Center for Advanced Materials Research, University of Sharjah, PO Box 27272, Sharjah, United Arab Emirates; 3 Dept. of Applied Physics, University of Sharjah, PO Box 27272, Sharjah, United Arab Emirates; 4 Dept. of Electrical Engineering, University of Sharjah, PO Box 27272, Sharjah, United Arab Emirates

## Abstract

Recent years have shown an increased interest in developing manufacturing processes for graphene and its derivatives that consider the environmental impact and large scale cost-effectiveness. However, today’s most commonly used synthesis routes still suffer from their excessive use of harsh chemicals and/or the complexity and financial cost of the process. Furthermore, the subsequent transfer of the material onto a substrate makes the overall process even more intricate and time-consuming. Here we describe a single-step, single-cell preparation procedure of metal-supported reduced graphene oxide (rGO) using the principle of bipolar electrochemistry of graphite in deionized water. Under the effect of an electric field between two stainless steel feeder electrodes, grapheme layers at the anodic pole of the wireless graphite were oxidized into colloidal dispersion of GO, which migrated electrophoretically towards the anodic side of the cell, and deposited in the form of rGO (*d*_(002)_ = 0.395 nm) by van der Waals forces. For substrates chemically more susceptible to the high anodic voltage, we show that the electrochemical setup can be adapted by placing the latter between the wireless graphite and the stainless steel feeder anode. This method is straightforward, inexpensive, environmentally-friendly, and could be easily scaled up for high yield and large area production of rGO thin films.

Graphene is a new class of planar *sp*^*2*^-hybridized carbon atoms in honeycomb lattice with exceptional properties: a room-temperature electron mobility of about 200000 cm^2^ V^−1^ s^−1 ^ [ref 1], 97.4% transmittance with as low as 125 Ωm^−1^ electrical sheet resistance^2^, a room-temperature thermal conduction of 5300 W m^−1^ K^−1^ [ref 3], a Young modulus of 1.0 TPa with 20% third-order elastic stiffness, a tensile strength of 130 GPa^4^, and a large theoretical specific surface area of 2630 m^2^ g^−1^ [ref 5]. Thus, graphene and graphene related materials have been suggested for numerous technological applications, including field effect transistors^6^, transparent electrodes^7^, single molecule detection^8^, biosensors^9^, and energy storage^10^, amongst many others^2^,^11^,^12^,^13^,^14^. In this regard, numerous synthesis routes have been reported to prepare the 2D carbon material, which can be categorized into two types: bottom-up and top-bottom approaches^15^. The bottom-up approaches include the chemical synthesis of graphene nanoribbons^16^ and graphene quantum dots^17^, epitaxial growth on SiC wafer^18^ and chemical vapor deposition on transition metals such as copper or nickel^19^. In contrast, the top-bottom category of methods-- that is the exfoliation of bulk graphite-- can be performed by mechanical cleavage^6^, or in a liquid media by sonication^20^, chemical oxidation-exfoliation-reduction^21^, and by intercalation-exfoliation^22^. The major drawbacks of most of these techniques are either a low production yield of graphene and graphene-based materials, and/or expensive multi-step chemical and physical processes, such as high temperature, ultra-high vacuum, or a series of time-consuming chemical oxidation/reduction reactions, making them less attractive for large scale manufacturing.

Nonetheless, the most commonly used approaches for graphite exfoliation remain chemical, yielding graphene oxide (GO), and require the use of strong oxidizing agents such as KMnO_4_, NaNO_3_ and KClO_3_ in concentrated acids such as H_2_SO_4_ and HNO_3_, with the generation of toxic NO_2_, N_2_O_2_ and ClO_2_^23^. GO is composed of not only *sp*^*2*^-hybridized carbon atoms, but it consists of ribbon-like structures of flat hexagons of carbon atoms, linked together by a network of cyclohexane chairs decorated by hydroxy, epoxy, ether, diol, and ketone groups^24^. Unlike graphene, which is not easily processable because of its insolubility in main solvents, GO is an electric insulator and intrinsically strongly hydrophilic, allowing it to be readily dispersed in many solvents for large scale and low cost preparation of thin films. This can be carried out for instance by dip coating^25^, spin coating^26^, electrophoretic deposition (EPD)^27^, and others^28^. With dip coating and spin coating, it is usually difficult to guarantee a preset thickness and uniformity of the deposit due to aggregation of GO. Whereas with EPD, Although the thickness in EPD could be controlled by adjusting the concentration, the zeta potential of the suspension, the deposition time, and the applied voltage, the carbonaceous material still ought to be prepared and dispersed in water or some organic solvents prior to deposition^27^. For example, An *et al*. reported the EPD of GO on conductive substrates to obtain reduced GO (rGO), but they had to first prepare a colloidal dispersion of the material by a modified Hummers’ method followed by a two-hour ultra-sonication process^29^.

In this work we present a straightforward single-step preparation method for metal-supported rGO using an isolated bipolar graphite rod in deionized water. The principle of bipolar electrochemistry consists on applying a static electric field between two feeder electrodes which sets in opposite polarizations of the wireless conductor (placed in between) with respect to the surrounding medium. Subsequently, the apparent difference of potential between its opposite ends would drive (if sufficient enough) simultaneous faradaic reactions of oxidation at the anodic pole, and reduction at the cathodic pole. This principle has been used for numerous applications, such as bulk manufacturing of dissymmetric Janus type objects^30^, establishing electrical contacts^31^, controlling the linear and rotational motion of microswimmers^32^, driving electrochemiluminescent reactions^33^, batch testing of electrocatalysts activity^34^ and corrosion behavior of materials^35^, amongst many others^36^. In the proposed bipolar electrochemical process, we combine in a single-cell and a single-step both the oxidation of the graphite rod yielding soluble GO, and the subsequent electrophoresis of the material and its deposition on a positively polarized substrate in a reduced form. Usually, the reduction of GO requires more chemical^37^, or electrochemical^14^, or thermal^38^ post-processing steps making the overall preparation of the material more complex and time-consuming. In addition to its simplicity and potential for large scale production, the merit of our method is that it is inexpensive, operates at ambient temperature, and is environmentally-benign because it does not require the use of any harsh chemicals.

## Results

**Figure 1:**
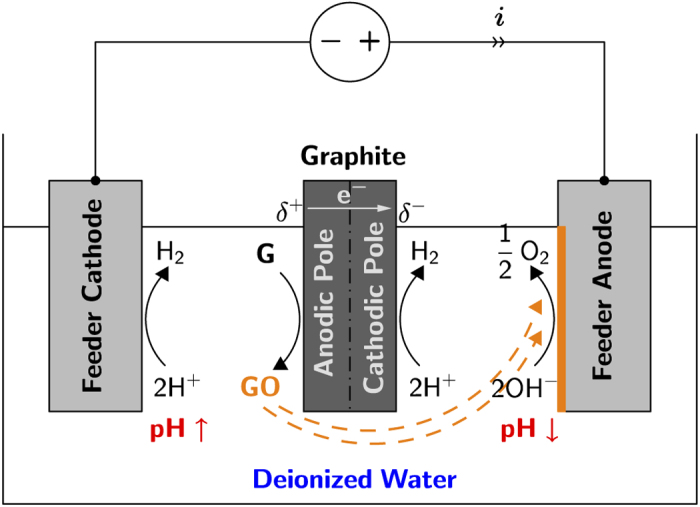
Schematic diagram of the bipolar electrochemical setup used for the preparation of stainless steel-supported rGO. In a typical synthesis process, a voltage difference is applied between two stainless steel feeding electrodes inducing a polarization of the wireless graphite. On the cathodic pole, water reduction reaction takes place, whereas GO, resulting from the oxidation of the anodic side of the graphite rod, migrates eletrophoretically towards the positive stainless steel to be deposited in the form a uniform light yellow thin film of rGO.

**Figure 2:**
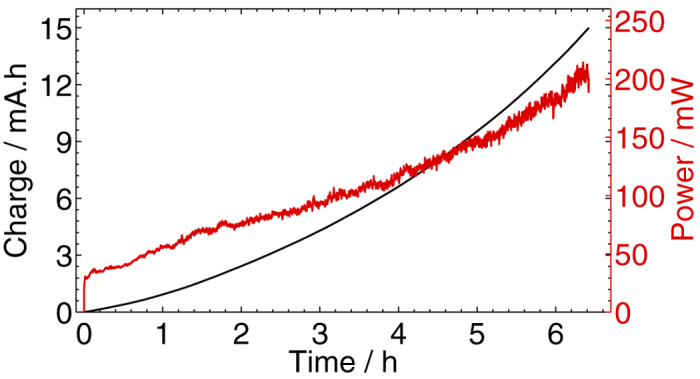
Evolution of total cell charge and power consumption vs. time for the preparation of stainless steel anode-supported rGO sample with a limiting charge of 15 mA.h. The applied cell voltage was 45 Vdc.

**Figure 3:**
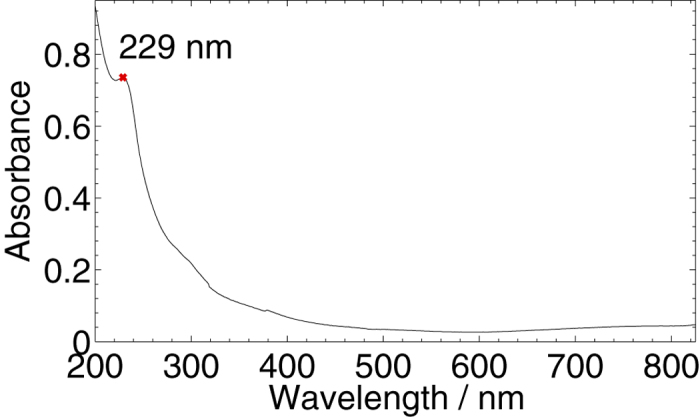
UV-vis light absorption spectrum of the the synthesis solution consisting of a stable colloidal dispersion of GO.

In Fig. 1 we show a schematic of the electrochemical setup used for the preparation of stainless steel-supported rGO. A static 15 V cm^−1^ electric field was set between two 1 × 2 cm^2^ stainless steel feeding electrodes placed 3 cm apart, with a floating graphite rod of 0.6 cm diameter positioned half-way in between. The solution used was deionized water of 18 MΩ cm resistivity. The two half reactions for water electrolysis took place: reduction of two water molecules at the stainless steel cathode to one molecule of gaseous hydrogen (2H^+^ + 2e^−^ → H_2_) and two hydroxyl ions (OH^−^), which under the effect of concentration gradient diffused towards the stainless steel anode to be discharged to half a molecule of oxygen gas and one molecule of water. Moreover, the electric field induced a maximum surface potential difference of 9 V (with the assumption that the potential drop is linear through the solution and negligible at the electrodes) between the two ends of the equipotential graphite substrate, which is sufficient enough (>1.5 V) to promote coupled redox reactions at the anodic and cathodic poles. Thus, it is the strength of the external electric field and the lateral length of the bipolar substrate that control this interfacial potential difference, as opposed to a standard two- or three-electrode configuration. We prepared several samples of stainless steel-supported rGO films by setting a limit on the cumulative total cell charge of 5, 10, …, 30 mA h. In Fig. 2, we show the time profiles of charge and power consumption measured for the sample prepared with 15 mA h charge. Similar trends were observed with other samples. The fluctuations in the power-time curve were the result of gas bubbles produced at and detached from the electrodes, and were visually observed. The distribution of the total cell current *i*_t_ (power *p*_t_ divided by 45 Vdc) between the solution ionic current (*i*_s_) and the bipolar electrode faradic current (*i*_bp_) is dependent on the relative resistances of the two media^39^. By writing *i*(*t*) = *i*_s_ + *i*_bp_, we can show that the ratio *i*_bp_/*i*_t_ is equal to *R*_s_/(*R*_s_ + *R*_bp_), with *R*_s_ and *R*_bp_ being the resistances of the solution and of the bipolar substrate respectively. Thus, a solution of high resistance would promote more faradic current through the floating graphite, which justifies the use of deionized water in this work as being a poor conductor. The total cell current increased with time as the solution conductivity increased with the course of faradic reactions proceeding at the lateral surfaces of the graphite substrate. The charge 
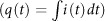
 vs time profile shown in Fig. 2 is nonlinearity increasing, which suggests that there was a positive feedback of charge into the system because of the continuous increase of the solution conductivity resulting from the oxidation of graphite into GO. For making copper electrical contacts^31^, or fabricating rectifying devices^40^, the bipolar electrochemical process consisted of electro-dissolving the sacrificial metal into its cationic precursors, and subsequently reducing them at the surface of a supporting substrate of opposite polarity in the form of wire contacts. Similarly here, negatively charged GO platelets from the depronation of carboxylate groups were generated at the anodic pole of the bipolar graphite where the pH is locally acidic (see Fig. 2)^41^. At eye view, the solution had a very light yellowish hue but still remained completely transparent and stable as a result of electrostatic repulsion of negative charges. The ultraviolet-visible (UV-vis) light absorption spectrum of the solution, shown in Fig. 3, depicts a typical absorbance peak for GO at 229 nm that is attributed to *π*–π* transitions of carbon-carbon double bond, and broad absorption in the visible light range. The broad shoulder centered around 300 nm is attributed to *n*–π* transitions of carbon-oxygen double bond^42^. The GO platelets were subsequently dragged parallel to the electric field by electrophoresis (the electrophoretic motion is defined by the net force resulting from the effects of the electric field on the negatively charged surface of GO, and on the counter-ions in the double-layer (retardation), the distortion of the double-layer (relaxation), and the viscous drag forces from the liquid^43^) and deposited on the surface of the positive electrode of the cell in the form of a coherent film, although at certain spots of the surface, the deposition was in competition with bubbles resulting from water splitting reaction. Contrary to other two- or three-electrode electrochemical configurations, because the sacrificial bipolar graphite acted as both anode and cathode simultaneously, this made the auxiliary anode free to accept a deposit (all in a single-step process). Deoxygenation of GO in the locally acidic environment (see Fig. 1) resulted in rGO, as it will be discussed in the characterization section below. Hasan *et al*. have showed through measurements of zeta potential that an increase of proton concentration, which was the case in the vicinity of the anode stainless steel in this work, reduced the electrostatic repulsion between GO sheets, and thus enabled their face-to-face layered deposition parallel to the substrate^44^. The adherence to the substrate surface was through coagulation of charged GO maintained by short range van der Waals attractive forces^28^,^45^. Although EPD has been successfully used for many types of deposits, the mechanism is still not completely understood^46^. Traditionally, the fundamental process that has been largely adopted in the literature is based on the Derjaguin-Landau-Verwey-Overbeek (DLVO) theory and the distortion of the particle double-layer under a dc electric field^45^. Other mechanisms have been proposed and discussed such as flocculation by particle accumulation, particle charge neutralization, electrochemical particle coagulation, electrical double layer distortion and thinning^45^. Each of these scenarios is valid in certain conditions and invalid in others^45^.

**Figure 4:**
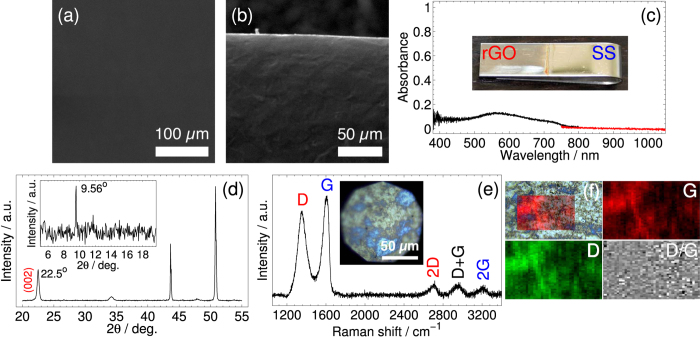
Morphological, optical, and structural characterization of stainless steel-supported rGO thin film obtained with 15 mA h cumulative charge: scanning electron micrograph at (**a**) the middle and (**b**) lower edge of the substrate, (**c**) Vis-NIR absorbance spectrum (inset shows a photograph of the stainless steel anode with rGO deposit), (**d**) XRD diffraction patterns, (**e**) micro-Raman spectrum obtained with *λ* = 514 nm laser excitation (inset shows the Raman microscopic image where the measurements have been carried out), and (**f**) micro-Raman map of the D, G and D-to-G peaks intensities over a selected surface area of 76 × 46 *μ*m^2^.

We characterized the surface of the stainless steel anodes-supported rGO prepared with 5, 10, …, 30 mA h by several microscopic, structural and optical techniques. We show the results obtained with 15 mA h charge as a typical example (the same conclusions hold for the other samples). Topological scanning electron micrographs from the center and the edge of the substrate are shown in Fig. 4(a,b). The center shows a completely flat surface of the deposit fully covering the depositing electrode, whereas at the edge of the substrate, a rug-like microstructure was observed with different heights. From the EDX two-dimensional map spectrum of the center of the electrode, we found 9.8 wt.% for carbon vs. 2.7 wt.% for oxygen. At the edge, the amount of carbon was higher to 15.9 wt.% vs. 2.9 wt.% oxygen. From the cross-sectional SEM studies of the stainless steel anode, we were unable to determine a sizable thickness for the deposits with any of the set charges (5, 10, …, 30 mA h), which suggests that it is in the nanometer range. This is probably due to the fact that, as the process took place at constant voltage, the deposition required steeper potential gradient at the solution/anode interface, which tends to flatten with increasing deposition as a result of increase of the local electric resistance^45^. Visible and near-infrared (Vis-NIR) spectral absorbance measurements of stainless steel anode-supported rGO are shown in Fig. 4(c). The absorbance is about 11.9% at 550 nm, and increased with the increase of deposited charge. The XRD patterns in Fig. 4(d) depict the typical rGO (002) diffraction line at 22.5° 2*θ* angle along with other characteristic lines for stainless steel. The corresponding interlayer spacing was found to be 0.395 nm, which is 18% higher than 0.335 nm of graphene’s in a single crystal graphite having true ABAB stacking^47^. GO’s interlayer spacing varies between 0.6 and 1.0 nm depending on the preparation method and the intercalated species^48^. This implies that there was partial restoration of graphitic structures by removal of intercalated, oxygen-containing functional groups once GO was electrophoretically deposited on the stainless steel anode. In the inset, showing the low angle XRD patterns, we did not record the GO diffraction line at 2*θ* = 11.3°, but a relatively low intensity (*ca.* 3% of the rGO (002) peak intensity), very sharp signal was detected at 9.56°, which corresponds to a long-range, highly crystalline structure of GO. This peak was short-lived and not found in all measurements. Dimiev and Tour studied the structural reaction of intermediate products in a modified Hummers method for graphite oxidation using micro-Raman spectroscopy and XRD^49^, and argued that this peak may originate from the originally-ordered graphene layers in bulk graphite. This signifies that in our bipolar electrochemical process, oxidized ordered graphene layers from the anodic pole of the sacrificial graphite rod remained intact in the course of their migration towards and deposition on the positive electrode of the cell. This is the rate-determining step of the process. In Fig. 4(e) we show a typical room temperature Raman spectrum collected from the stainless steel supported-rGO film with a laser excitation wavelength of *λ* = 514 nm. The inset is an optical image of the sample taken with the Raman microscope imaging system, and depicts opalescent patches of moss green and sky blue colors. Raman spectra from these two different regions showed differences only in the intensities of Raman peaks (see maps of D, G and D-to-G peaks intensities in Fig. 4(f) measured over a 76 × 46 *μ*m^2^ surface area). The spectrum consists of a set of distinct peaks with two prominent and broad D and G peaks at 1353 and 1604 cm^−1^ respectively, and three low intensity and broad 2D, D+G, and 2G peaks at higher wavenumbers^28^. The D peak is ascribed to the *A*_1*g*_ breathing modes of carbon atoms aromatic rings that are induced by defects, and the G peak originates from the *E*_2*g*_ Raman allowed optical phonon at the Brillouin zone center due to bond stretching of carbon atom pairs in both rings and chains. The D to G peak intensity ratio, which is a measure of degree of disorder and is inversely proportional to the average lateral size of the *sp*^2^ clusters, was found to be 0.85 as reported elsewhere^50^. Second-order Raman scattering denoted by 2D is observed at 2707 cm^−1^ (approximately twice the energy of the D mode). The D+G and 2G are observed at 2956 and 3205 cm^−1^, respectively.

**Figure 5:**
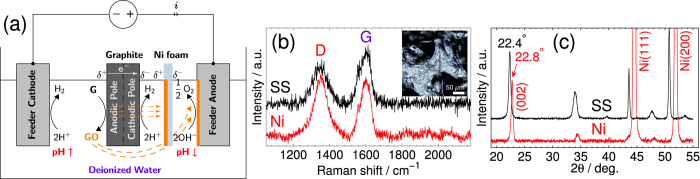
(**a**) Schematic of a modified bipolar electrochemical setup used for the preparation of Ni foam-supported rGO. Micro-Raman spectra and XRD patterns for both Ni foam and stainless steel feeder anode are shown in (**b**,**c**), respectively; the respective interlayer spacings from the (002) diffraction peaks were found to be 0.390 and 0.396 nm.

We carried out further experimental trials using the same setup and settings described in Fig. 1, but with a 0.2 mm-thick Ni foam anode (1 × 2 cm^2^) instead of the stainless steel plate. Because of the high electrical stresses on the Ni anode we were not able to identify the presence of rGO on its surface. The solution turned greenish in color after a certain time resulting from the electro-dissolution of Ni into Ni^2+^. However, by adapting the electrochemical configuration as depicted in Fig. 5(a) (see electric characteristics in SI Fig. 1), in which both the sacrificial graphite rod and Ni foam were wirelessly suspended between the two stainless steel auxiliary electrodes, we were able to confirm by Raman spectroscopy and XRD (see Fig. 5(b,c)) the deposition of rGO on both Ni foam and stainless steel anode. In this situation, the applied voltage was set to 30 Vdc (i.e. *ca.* 10 V cm^−1^ electric field) inducing a low 0.2 V apparent potential difference across the lateral surfaces of Ni foam, which is not high enough to initiate its electro-dissolution reaction. Nonetheless, the substrate is divided into a cathodic pole (*δ*^−^), and an anodic pole (*δ*^+^) on which the EPD of rGO took place. The deposition of rGO on Ni foam confirms that it proceeded through van der Waals forces, and not electrochemically.

## Discussion

In summary, we demonstrated an easy, single-step, single-cell route towards the development of transparent rGO thin films on conductive substrates using bipolar electrochemistry. The electrochemical methods for the synthesis of graphene and graphene-based materials, whether using two- or three-electrode cells, stand out as the ones that require less processing time, operate at ambient conditions, and with minimum instrumental and operational costs. But contrary to conventional electrochemical processes where the anode and cathode are physically separated, the graphite substrate in our bipolar electrochemical setup acted as both anode and cathode at the same time, allowing its oxidation into GO on the anodic side and hydrogen production on the cathodic side to take place. Subsequently, GO was electrophretically drawn to and deposited on the anode side of the cell by van der Waals forces in the form of rGO. We also showed that EPD of rGO can also take place at the anodic pole of another floating conductive substrate (Ni foam was taken as an example) that was placed between the graphite and the auxiliary anode. The kinetics of the EPD process and the amount of deposit are mainly functions of the time-dependent concentration and average velocity of charged GO, the zeta potential of the colloidal suspension, as well as the distribution of potentials and resistances in the electrochemical cell^45^. Further investigations are required to elucidate the rates at which the amount and thickness of the deposit evolve.

This approach, from the formation of GO dispersion to their deposition and reduction to rGO, took place at room temperature in deionized water and could be readily scaled-up for industrial applications. In principle, the bipolar process we illustrated here offers great flexibility that would make it possible to fabricate coatings on flat or complex surfaces and on porous three-dimensional structures that can serve as ideal platforms for cutting-edge optoelectronics, energy, and sensing applications.

## Methods

### Synthesis of Metal-supported Reduced Graphene Oxide

A schematic of a typical synthesis setup of metal-supported rGO using bipolar electrochemistry is shown in Fig. 1. It consists of two 304 L stainless steel feeding electrodes (1 × 2 cm^2^), placed 3 cm apart in deionized water (initially of 18 MΩ cm resistivity at 25 °C), connected to a 48 V/1 A booster channel of a BioLogic VSP 300 potentiostat. The applied voltage was set to 45 Vdc. A 0.6 cm diameter graphite rod was positioned wirelessly between the two stainless steel electrodes. For substrates more prone to electro-dissolution under anodic voltage, a second setup was used as shown in Fig. 5(a), where the depositing substrate (Ni foam of 0.2 mm thickness) is wirelessly placed between the graphite and the stainless steel anode. The applied voltage was 30 Vdc in this situation.

### Characterization

The morphological characterization of metal-supported rGO was performed using a TESCAN VEGA3 XM scanning electron microscope (SEM) equipped with an energy-dispersive X-ray (EDX) detector. XRD patterns were recorded in the 2*θ* geometry at 0.02° 2*θ* step increment with a Bruker D8 Advance X-ray diffractometer (Cu K_α_ radiation; *λ* = 1.5406 *Å*, 40 keV, 40 mA). The Raman spectra were measured at room temperature in the back scattering configuration with a confocal Renishaw inVia Raman microscope equipped with visible Ar^+^ 514.5 nm (2.41 eV) wavelength laser. The power was 1 mW on a spot size of *ca.* 1 *μ*m^2^ with 50× objective. The fluorescence background was subtracted from the original data. The ultraviolet-visible (UV-vis) light absorption spectrum of the solution was acquired using a Perkin-Elmer Lambda 800 spectrometer over the wavelength range 200–900 nm. Visible and near-infrared (Vis-NIR) absorbance measurements were acquired with two Ocean Optics Maya2000 Pro spectrometers, which together cover the range of 380–1100 nm wavelengths. The sample was placed on the aperture of an Ocean Optics ISP-REF integrating sphere equipped with built-in tungsten-halogen light source (spectral range: 360–2500 nm).

## Additional Information

**How to cite this article**: Allagui, A. *et al*. Reduced Graphene Oxide Thin Film on Conductive Substrates by Bipolar Electrochemistry. *Sci. Rep.* **6**, 21282; doi: 10.1038/srep21282 (2016).

## Supplementary Material

Supplementary Information
