# Erratum: Reduced Graphene Oxide Thin Film on Conductive Substrates by Bipolar Electrochemistry

**DOI:** 10.1038/srep26864

**Published:** 2016-06-02

**Authors:** Anis Allagui, Mohammad Ali Abdelkareem, Hussain Alawadhi, Ahmed S. Elwakil

Scientific Reports 6: Article number: 21282;10.1038/srep21282 Published online: 02 17 2016; Updated: 06 02 2016

The original version of this Article contained errors in the spelling of the authors Anis Allagui, Mohammad Ali Abdelkareem, Hussain Alawadhi and Ahmed S. Elwakil which were incorrectly given as Allagui Anis, Ali Abdelkareem Mohammad, Alawadhi Hussain and S. Elwakil Ahmed respectively.

These errors have now been corrected in the PDF and HTML versions of the Article.

