# Supplementary material for: Reduced Graphene Oxide Thin Film on Conductive Substrates by Bipolar Electrochemistry
Source: Sci Rep. 2016 Feb 17;6:21282. doi: 10.1038/srep21282 (PMC4756714; doi:10.1038/srep21282)
Supplement: Supplementary Information [file srep21282-s1.pdf]

Reduced Graphene Oxide Thin Film on Conductive Substrates by Bipolar Electrochemistry

Anis Allagui[1,2,*], Mohammad Ali Abdelkareem[1], Hussain Alawadhi[3,2], and Ahmed S. Elwakil[4]

[1]Dept. of Sustainable and Renewable Energy Engineering, University of Sharjah, PO Box 27272, Sharjah, United Arab Emirates
[2]Advanced Materials Research Center, University of Sharjah, PO Box 27272, Sharjah, UAE
[3]Dept. of Applied Physics, University of Sharjah, PO Box 27272, Sharjah, United Arab Emirates
[4]Dept. of Electrical Engineering, University of Sharjah, PO Box 27272, Sharjah, United Arab Emirates
[*]aallagui@sharjah.ac.ae

SUPPORTING INFORMATION

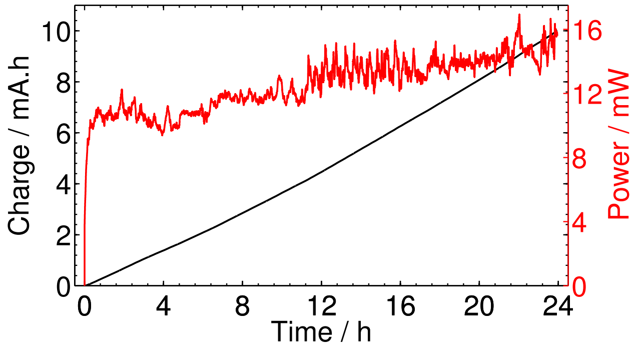

Figure 1. Evolution of total cell charge and power consumption vs. time for the preparation of Ni foam-supported rGO sample using the electrochemical setup depicted in figure 5(a)). The applied cell voltage was 30 Vdc with a limiting charge of 10 mA.h
